# Occupational Hazards, Social Support, and Quality of Working Life in Sub-District Health Promoting Hospitals in Southern Thailand: A Cross-Sectional Study

**DOI:** 10.3390/ijerph23020272

**Published:** 2026-02-23

**Authors:** Sasithorn Thanapop, Sintira Lucksila, Nattachalisa Saritdisuk, Warangkana Chankong, Linxiong Wu, Chamnong Thanapop

**Affiliations:** 1 School of Health Science, Sukhothai Thammathirat Open University, Nonthaburi 11120, Thailand or sasilau@gmail.com (S.T.); warangkana.cha@stou.ac.th (W.C.); 2 Khaopun Sub-District Health Promoting Hospital, Trang 92130, Thailand; sintira1@gmail.com; 3 School of Public Health, Walailak University, Nakhon Si Thammarat 80160, Thailand; bamza23@gmail.com; 4 School of Public Health, Kunming Medical University, Kunming 650500, China; wulinxiong@kmmu.edu.cn; 5 Excellent Center for Public Health Research, Walailak University, Nakhon Si Thammarat 80160, Thailand

**Keywords:** healthcare personnel, occupational hazards, primary healthcare, social support, work-related quality of life

## Abstract

**Highlights:**

**Public health relevance—How does this work relate to a public health issue?**

- This study examines occupational health and quality of working life (QWL) among frontline personnel in Thailand’s community-based primary healthcare system, where healthcare delivery is inherently community-engaged.
- It explores how community-embedded social support and occupational hazard exposure interact within decentralized sub-district health promoting hospitals (SHPHs).

**Public health significance—Why is this work of significance to public health?**

- Findings demonstrate that social support—extending beyond the workplace to community networks—is the strongest determinant of QWL, highlighting a key mechanism within community-engaged health systems.

**Public health implications—What are the key implications or messages for practitioners, policy makers and/or researchers in public health?**

- Practitioners and health systems require a dual approach for increasing organizational social support and systematically mitigating occupational risks—particularly biological and chemical hazards—to ensure a resilient and sustainable community health system.
- Policy makers and researchers can use this data to optimize working hours and compensation structures, ensuring a sustainable workforce amidst the ongoing transition of health services to local administrative organizations.

**Abstract:**

Healthcare personnel working in sub-district health promoting hospitals (SHPHs) are vital to Thailand’s primary healthcare system but often face occupational, psychosocial, and organizational challenges that may affect their quality of working life (QWL). This study aimed to assess QWL and identify its key predictors among SHPH healthcare personnel in southern Thailand. A cross-sectional survey was conducted among 340 healthcare personnel in Nakhon Si Thammarat Province using stratified random sampling. Data were collected through a structured questionnaire covering socio-demographic characteristics, working conditions, occupational hazard exposures, social support, and QWL measured by the 36-item Thai version of the QWL scale. Descriptive statistics and stepwise multiple linear regression analyses were performed. Participants were predominantly female (80.9%) with a mean age of 34.0 years (SD = 9.2), and one-third (33.2%) worked more than eight hours per day. Most participants (75.6%) had moderate hazard exposure, while 73.2% reported high social support. Overall, 51.2% of respondents had good QWL, with safe and healthy working conditions and social relevance of work life rated highest. Regression analysis identified social support (β = 0.790, *p* < 0.001) and working hours per day (β = 0.109, *p* = 0.001) as positive predictors, while work experience (β = −0.064, *p* = 0.049) was a negative predictor (R^2^ = 0.655). These findings emphasize the need for organizational strategies that strengthen social support and effectively manage working conditions, including the organization of working hours, to promote sustainable quality of working life among healthcare personnel in Thailand’s primary healthcare system.

## 1. Introduction

The global healthcare system is currently facing a critical shortage of healthcare workers, posing a substantial challenge to the achievement of Universal Health Coverage (UHC) and the United Nations’ Sustainable Development Goals (SDGs) [1,2]. In response to rising healthcare demands and resource constraints, many countries have pursued decentralization reforms in health system governance. However, decentralization is increasingly recognized as a hybrid process, in which local autonomy is balanced with central coordination, regulation, and equity oversight, particularly in low- and middle-income countries (LMICs) [3,4,5].

Within this evolving governance context, Primary Healthcare (PHC) has become the cornerstone of health service delivery, emphasizing community-based prevention, health promotion, and continuity of care. The World Health Organization underscores that the resilience and effectiveness of PHC systems are closely linked to the well-being and quality of working life (QWL) of frontline healthcare personnel [6]. In many LMICs, including Thailand, healthcare personnel operate in resource-constrained environments while balancing clinical responsibilities with expanding administrative duties and routine engagement with communities, local authorities, and civil society actors [7,8].

In Thailand, Sub-district Health Promoting Hospitals (SHPHs) constitute the frontline infrastructure of the national PHC system. SHPH personnel—including public health practitioners, nurses, and technical officers—provide preventive, promotive, and rehabilitative services through both facility-based care and community outreach activities, such as home visits, disease surveillance, and collaboration with village health volunteers and local administrative organizations. While such community-engaged practice is fundamental to PHC effectiveness, it may also increase exposure to occupational hazards, including biological risks, ergonomic strain, and psychosocial stressors, particularly in decentralized and post-pandemic health systems [9,10,11].

Quality of Working Life is a multidimensional psychological and organizational construct reflecting the extent to which employees’ work environments satisfy both professional aspirations and fundamental human needs [12,13]. Grounded in Walton’s eight-component framework, QWL encompasses adequate and fair compensation, safe and healthy working conditions, opportunities for skill development and career growth, social integration in the workplace, constitutionalism, work–life balance, and the social relevance of work. Extensive evidence demonstrates that compromised QWL is associated with adverse outcomes such as occupational stress, burnout, absenteeism, and workforce turnover, whereas higher QWL supports productivity and organizational commitment [14,15,16].

Within this framework, social support represents a key psychosocial resource that can buffer occupational stress and enhance resilience. According to House’s conceptualization, social support encompasses emotional, informational, instrumental, and appraisal dimensions that help individuals cope with job demands [17]. In community-based PHC systems, social support may extend beyond formal workplace relationships and become embedded within routine interactions with community members, volunteers, and local networks, reinforcing professional identity and perceived work meaningfulness [18,19,20].

Despite growing recognition of occupational hazards and psychosocial challenges in healthcare, empirical research examining the interplay between occupational exposures, social support, and QWL among primary healthcare personnel in community-engaged settings remains limited, particularly in LMIC contexts. Most existing studies have focused on hospital-based nurses in urban settings, leaving an evidence gap regarding decentralized and community-embedded health workforces such as SHPH personnel in Thailand. Moreover, limited post–COVID-19 evidence exists on how occupational risks and social support jointly influence QWL within PHC systems undergoing governance and workload transitions.

Therefore, this study aims to assess the level of QWL and identify its key predictors among healthcare personnel working in SHPHs in southern Thailand, with particular attention to sociodemographic characteristics, occupational hazard exposures, social support, and work experience. By situating these factors within a community-engaged PHC context, this study contributes new evidence on workforce sustainability in decentralized primary healthcare systems and informs occupational health and workforce support strategies in LMIC settings.

## 2. Materials and Methods

### 2.1. Study Design and Setting

This analytic cross-sectional study was conducted in Nakhon Si Thammarat Province, the most populous province in Southern Thailand, during November and December 2023. The study population comprised 2988 healthcare personnel working in 244 SHPHs across the province. The required sample size was calculated using a finite population sample size formula, commonly applied in cross-sectional health studies, assuming a 95% confidence level and a 5% margin of error, as described in standard biostatistics literature [21]. Based on the total population size, the minimum required sample size was approximately 340 participants.

SHPHs operate as community-based primary healthcare facilities within Thailand’s decentralized health system. Healthcare personnel working in SHPHs routinely provide preventive services, health promotion, disease surveillance, and emergency response activities through direct engagement with community members, village health volunteers, local administrative organizations, and community leaders. These activities require personnel to perform their duties both within healthcare facilities and in household and community settings, reflecting the routine working conditions of SHPH personnel.

Eligible participants were healthcare personnel who had been working in their current position for at least one year. Stratified random sampling was applied to recruit participants. Exclusion criteria included incomplete questionnaire responses and voluntary withdrawal from the study.

### 2.2. Data Collection and Measurements

Data were collected using a structured four-part questionnaire. The first section assessed sociodemographic characteristics, including gender, age, marital status, educational attainment, presence of non-communicable diseases (NCDs), monthly income, and work experience, defined as the number of years working in the current job role within the primary healthcare system. Work experience was measured as a self-reported continuous variable (years) and grouped into 1–5 years, 6–10 years, and more than 10 years for descriptive presentation (Table 1). In this setting, working more than 8 h per day typically reflects compensated shift duties, such as early, late, or weekend shifts, rather than excessive uncontrolled workload.

The second section assessed working conditions and occupational hazard exposures using a 12-item checklist covering five domains: physical (3 items), biological (2 items), chemical (2 items), ergonomic (2 items), and psychosocial hazards (3 items). Items reflected common occupational risks in primary healthcare settings, such as inadequate lighting and ventilation (physical), contact with excreta or wound drainage (biological), use of disinfectants or chemicals (chemical), prolonged standing (ergonomic), and work–rest imbalance or limited support (psychosocial). All items were rated on a 5-point Likert scale (1 = “not at all” to 5 = “extremely”). Domain-specific scores were calculated by summing the items within each hazard domain, resulting in different possible score ranges according to the number of items per domain. For descriptive analysis, domain-specific scores were categorized into low, moderate, and high exposure levels using the class-interval method, based on the theoretical score range of each domain [9,22]. The percentages reported in Table 2 reflect the distribution of participants across exposure levels within each hazard domain.

The third section measured social support based on House’s conceptual framework [17], using an 8-item scale assessing support from family, friends, colleagues, and organizations. Responses were rated on a 5-point Likert scale. Total scores ranged from 8 to 40 and were categorized into low (8–18), moderate (19–29), and high (30–40) levels using the class-interval method, consistent with previous studies [18,19]. The final section assessed QWL using a 36-item scale based on Walton’s framework [12,13,23]. It evaluates eight dimensions, with scores classified as poor, moderate, or good using the class-interval method.

The questionnaire’s validity was confirmed by three experts (IOC = 0.67–1.00), and its reliability was confirmed using Cronbach’s alpha for Section 2, Section 3 and Section 4, with coefficients of 0.87, 0.93, and 0.96, respectively. Data collection was conducted by mailing questionnaires with an information sheet and written informed consent form. Participants were informed of the study objectives, voluntary participation, confidentiality, and the right to withdraw at any time without consequences. A total of 340 completed questionnaires that met the inclusion criteria were obtained and included in the final analysis, indicating a high level of participation.

### 2.3. Statistical Analysis

Data were analyzed using SPSS version 23.0 (IBM Corp., Singapore). Descriptive statistics were used to summarize participant characteristics and variable scores. Domain-specific occupational hazard exposures were reported descriptively to reflect distinct dimensions of working conditions, whereas an overall occupational hazard exposure score, derived from all 12 items, was used as a composite predictor in the inferential analysis.

Bivariate associations between independent variables (sociodemographic characteristics, working conditions, occupational hazard exposure, and social support) and QWL were examined using Pearson’s product–moment correlation or point-biserial correlation, as appropriate. Variables showing potential associations with QWL (*p* < 0.25) were entered into a stepwise multiple linear regression model to identify significant predictors. Gender and age were included in the final model as control variables. Model assumptions of normality and multicollinearity were assessed prior to analysis. Statistical significance was set at *p* < 0.05.

## 3. Results

### 3.1. Socio-Demographics and Working Conditions of Healthcare Personnel in SHPH

A total of 340 healthcare personnel participated in this study, most of whom were female (80.9%). Participants were aged 22–60 years, with 40.6% aged under 30 years and 34.1% aged 30–39 years. More than half were single (52.4%). Nearly all participants held at least a bachelor’s degree or higher (97.1%). Regarding health status, 13.5% reported having NCDs. In terms of job position, the largest group were public health practitioner (56.2%), followed by public health technical officers (17.6%), registered nurses (15.9%), nurse practitioners (7.6%), and dental public health officers (2.6%). For income, the majority (60.9%) earned between 15,000 and 30,000 Thai baht per month, while 28.2% earned more than 30,000. Of the participants, 41.5% reported 1–5 years of work experience, 23.8% had 6–10 years, and 34.7% had more than 10 years of experience. In terms of working hours, 33.2% of participants reported working more than 8 h per day (Table 1).

### 3.2. Occupational Hazard Exposures and Social Support Among Healthcare Personnel in SHPH

Healthcare personnel in SHPHs reported varying levels of occupational hazard exposures and social support (Table 2). Most participants (*n* = 257; 75.6%) were categorized as having moderate overall hazard exposure, while 83 (24.4%) had low exposure. Among hazard types, physical exposure was predominantly low (75.0%), whereas biological exposure showed the highest proportion of high-level exposure (47.6%), followed by chemical exposure (35.3%). Ergonomic exposure was mostly moderate (89.1%), and psychosocial exposure was largely low (62.1%). Regarding social support, 248 participants (73.2%) reported high support and 91 (26.8%) reported moderate support.

### 3.3. QWL of Healthcare Personnel in SHPH

The QWL scores indicated generally favorable outcomes, although variation was observed across domains (Table 3). Overall, more than half of respondents reported good QWL (51.2%), while 45.6% had moderate levels and only 3.2% reported poor QWL. In terms of specific domains, adequate and fair compensation emerged as a notable challenge, with more than half of participants reporting poor (27.6%) or moderate (29.7%) levels. Similarly, work–life balance reflected limitations, with 2.9% classified as poor and one-third (33.5%) moderate. By contrast, other domains showed relatively strong results. Safe and healthy working conditions were rated positively, with 75.9% reporting good QWL. Likewise, high proportions of respondents reported good levels in social integration in the workplace (72.9%), opportunities to use and develop human capacities (70.6%), and opportunities for continued growth and security (65.0%). Constitutionalism in the workplace and social relevance of work life were also perceived as favorable, with 67.9% and 75.3% rating them as good, respectively.

### 3.4. Key Predictors of QWL

Bivariate associations among all study variables were examined prior to regression analysis. The correlation matrix, including QWL, focal predictors, and sociodemographic covariates, is presented in Table S1 and was used to evaluate bivariate relationships and screen for potential multicollinearity.

In the stepwise multiple linear regression analysis, social support emerged as the strongest positive predictor of QWL (β = 0.790, *p* < 0.001). Working hours per day were also positively associated with QWL (β = 0.109, *p* = 0.001), whereas work experience showed a small but statistically significant negative association (β = −0.064, *p* = 0.049). The final model met assumptions of multicollinearity (VIF < 2) and overall model adequacy. The composite occupational hazard exposure score was not retained as a significant predictor in the final regression model. Overall, the final regression model explained 65.5% of the variance in QWL (R^2^ = 0.655), indicating substantial explanatory power (Table 4).

## 4. Discussion

### 4.1. Socio-Demographic and Occupational Characteristics

This study revealed that the majority of healthcare personnel in sub-district health promoting hospitals (SHPHs) were young to middle-aged females, consistent with previous reports indicating that the Thai primary healthcare workforce is predominantly female and under 40 years of age [6]. Most participants held at least a bachelor’s degree and were employed as public health practitioners, reflecting the professionalization of Thailand’s primary healthcare workforce. The mandated staffing ratio and functional delineation for SHPH personnel is set at approximately 1 registered nurse to 2 public health practitioners and 1 auxiliary staff member (1:2:1). The aggregate staffing ratio of active personnel per 1000 residents is recorded at 3.1 [24].

Although most participants reported working within standard hours, approximately one-third worked more than eight hours per day, suggesting notable workload pressure similar to findings among community health workers in decentralized primary healthcare settings in low- and middle-income countries [25]. The relatively low prevalence of non-communicable diseases (13.5%) indicates generally good health status. Furthermore, modest income levels may influence job satisfaction and perceived fairness at work [14]. As previously stated, this study was conducted following the widespread COVID-19 pandemic in Thailand. Community-level healthcare personnel were actively engaged in proactive preventative and control services. Moreover, it is important to note that beyond routine healthcare provision, SHPH personnel are burdened with various additional responsibilities, such as administrative functions, strategic and project-based planning, annual action plans, emergency protocols, and ad hoc duties. These are further compounded by obligations related to staff training, research and innovation development, and supervision, monitoring, and evaluation activities. Although the majority of the staff maintained good health, the observed workload and duties, including personnel-to-population ratio necessitates strategic planning to ensure an optimal working environment.

Such community-engaged practice may intensify role demands and administrative burden, particularly in resource-constrained settings undergoing governance transitions. These findings underscore the importance of considering occupational context when interpreting quality of working life (QWL) among primary healthcare personnel.

### 4.2. Occupational Hazard Exposures and Social Support

Occupational hazard exposure among SHPH personnel was moderate overall, with biological and chemical hazards being the most prevalent. These findings are consistent with previous studies reporting frequent exposure to infectious agents and disinfectants in primary healthcare settings [9,10,11]. Ergonomic risks, including prolonged standing and patient handling, were also commonly reported, aligning with evidence from nursing studies on musculoskeletal strain [26]. Although Thailand has implemented Occupational and Environmental Medicine Service Standards for SHPHs since 2014, including hazard surveillance, risk management, health examinations, immunization, and safety standard operating procedures [27,28], the persistence of moderate hazard exposure suggests that continuous and effective implementation remains essential in community-based primary care settings.

Despite these occupational challenges, the majority of participants reported high levels of social support. Within the Thai primary healthcare context, strong collaboration between healthcare personnel, community members, village health volunteers, and local leaders is deeply embedded in routine public health practice, as demonstrated during the COVID-19 prevention and response efforts [29]. This supportive social environment may help reinforce work motivation and sustain performance under demanding conditions. Consistent with previous evidence, strong social support has been shown to buffer occupational stress, enhance resilience, and improve morale among healthcare workers [18,30,31].

Importantly, social support within SHPHs appears to function as a community-embedded psychosocial resource, extending beyond formal workplace relationships. Routine engagement with community actors may strengthen emotional, informational, and instrumental support, thereby mitigating stress associated with community-based service delivery. This community engagement not only shapes occupational risk exposure but also provides an informal yet powerful protective mechanism against burnout, supporting workforce retention and sustainability in rural primary healthcare systems [8,32]. This finding highlights a distinctive feature of community-engaged primary care, where close social ties may simultaneously increase job demands while also providing protective psychosocial resources.

### 4.3. Quality of Working Life

Overall, the QWL among SHPH healthcare personnel ranged from moderate to good. Most participants reported positive perceptions in domains such as safe and healthy working conditions and social relevance of work life. These findings suggest that healthcare personnel perceive their work as meaningful and socially valuable, consistent with Walton’s (1974) framework [12], which emphasizes the importance of workplace safety and the social contribution of work to overall QWL.

In contrast, the domains of adequate and fair compensation and work–life balance received the lowest scores. This pattern reflects persistent challenges related to limited remuneration and workload imbalance, which have been widely reported in primary healthcare and rural health settings [11,33]. Despite these structural constraints, SHPH personnel appear to maintain a relatively positive overall QWL, potentially driven by intrinsic motivation and a strong sense of professional purpose. Similar findings have been observed in other studies, indicating that professional commitment and internalized values may partially offset dissatisfaction with extrinsic working conditions [34].

The high ratings for the social relevance of work life domain may be partly explained by the community-engaged nature of SHPH practice, in which healthcare personnel develop close and sustained relationships with the populations they serve. Such community engagement may strengthen professional identity, reinforce intrinsic motivation, and enhance perceived work meaningfulness, even in resource-limited contexts. However, this embeddedness within the community also presents potential challenges. Without adequate organizational support, community engagement may increase emotional labor, role overload, and work–life imbalance, highlighting the dual nature of community-based healthcare work. These findings underscore the need for organizational and policy-level strategies that not only preserve the meaningfulness of community-engaged practice but also address structural determinants of QWL, particularly compensation adequacy and workload management.

### 4.4. Predictors of Quality of Working Life

The stepwise multiple linear regression analysis revealed that three key variables—social support, daily working hours, and work experience—significantly predicted the Quality of Work Life (QWL) among primary healthcare personnel, accounting for 65% of the total variance. The most potential predictor identified in this study was social support, the strongest positive predictor of QWL. The prominent role of social support is consistent with the Job Demands–Resources (JD–R) framework, in which social support functions as a key job resource buffering work-related stress in high-demand primary healthcare settings. Prior evidence shows that supportive relationships and cohesive networks enhance job satisfaction, resilience, and psychological well-being among healthcare workers [15,18,19,35], including during the COVID-19 pandemic when social connectedness protected against burnout and mental health decline [2,15,35,36]. From a community-engaged perspective, social support may represent a mechanistic pathway through which routine community engagement influences QWL.

In Thailand’s decentralized primary healthcare system, sustained interaction with community networks can foster trust, shared responsibility, and collective problem-solving. In this sense, social support in SHPHs extends beyond interpersonal workplace relationships and is embedded within everyday community-based practice, reflecting the collectivist resilience characteristic of many Asian healthcare systems. While excessive workload is traditionally viewed as a detriment to well-being, within the specific context of the Thai healthcare system, longer hours may be intrinsically linked to increased financial incentives or a heightened sense of professional commitment. This finding should be interpreted cautiously, as longer working hours in this context often reflect compensated overtime or structured shift duties rather than purely increased workload intensity, and the positive association may therefore be driven by incentive structures rather than work strain alone. As suggested by Sirisawasd et al. (2014) [37], when increased workload is accompanied by adequate compensation and recognized professional value, it may contribute to higher levels of work satisfaction rather than immediate burnout. Conversely, work experience was found to be a negative predictor of QWL. This inverse relationship suggests that as healthcare workers progress in their careers, they may face cumulative emotional exhaustion or a “career plateau”. This aligns with the “Work Ability” framework proposed by Ilmarinen (2019) [38], which posits that without age-specific work adjustments and continuous workplace health promotion, the perceived quality of life in the workplace tends to decline as employees age and their tenure increases. In Thailand, the negative association between work experience and QWL may reflect cumulative administrative burden, limited career progression, and increasing role expectations among senior personnel in community-based health systems. In the SHPH context, experienced staff often shoulder a dual burden of maintaining clinical standards while navigating administrative transitions under Local Administrative Organizations, a pattern also linked to occupational fatigue and burnout in previous studies [2,14,39]. Together, these findings indicate that sustaining QWL requires integrated workforce strategies that combine community-derived psychosocial support with equitable compensation, strengthened supervision, and clearer career pathways, particularly in decentralized primary healthcare systems.

### 4.5. Implications and Recommendations

The findings of this study suggest that community-engaged primary healthcare practice has a dual influence on quality of working life. Healthcare authorities in Southern Thailand should prioritize the institutionalization of robust social support systems within SHPHs as the primary driver for enhancing QWL, while ensuring that working hours are strategically managed to align with equitable incentives and professional well-being. Furthermore, the implementation of age-management strategies is essential to address the decline in work-related well-being among senior personnel through targeted job redesign and the transition of experienced staff into consultative roles. Integrating these organizational support mechanisms with age-specific human resource management, in accordance with the work ability framework, will foster a resilient and sustainable primary care workforce capable of meeting the unique healthcare demands of the region.

### 4.6. Limitations

This study has limitations. First, as an analytic cross-sectional survey conducted in Nakhon Si Thammarat Province, the findings reflect one time point and cannot establish temporal order or causality, and generalizability beyond SHPHs in this setting may be limited. Second, data were obtained through a mailed, self-administered questionnaire, so recall and social-desirability bias. Although stratified random sampling was employed to enhance representativeness, the exclusion of incomplete questionnaires and personnel with less than one year of work experience may have introduced selection bias. Third, occupational hazard exposure, social support, and QWL were based on Likert-scale self-ratings and categorized using the class-interval method; these subjective classifications may not capture objective exposure intensity or nuanced differences between participants. Although stratified multi-stage sampling was applied, eligibility criteria and exclusion of incomplete responses may have introduced selection bias. The relatively high R^2^ observed in the regression model may partly reflect shared measurement context resulting from self-reported instruments, which could inflate associations among psychosocial variables. Finally, predictors were selected using stepwise multiple linear regression; although assumptions were checked, stepwise procedures can be sample-dependent, and residual confounding from unmeasured organizational factors may remain. The positive association between working hours and QWL may reflect contextual compensation structures in SHPHs and therefore may not be generalizable to primary healthcare settings where extended working hours are not accompanied by overtime incentives.

## 5. Conclusions

Healthcare personnel in Southern Thai SHPHs reported moderate-to-high QWL, predominantly driven by social support—the strongest positive predictor—and daily working hours, while longer work experience was associated with lower QWL. These findings underscore the central role of psychosocial and organizational resources within Thailand’s decentralized primary healthcare system. In this community-engaged context, social support from colleagues, supervisors, and local networks functions as a critical buffer against occupational demands and hazards, reinforcing resilience and work meaningfulness. Strengthening structured community-based support mechanisms may therefore serve as both a protective psychosocial strategy and a sustainable workforce retention approach.

## Figures and Tables

**Table 1:** Socio-demographics of healthcare personnel in SHPHs (*n* = 340).

Characteristics	*n*	%
Gender		
Male	65	19.1
Female	275	80.9
Age (year)		
<30	138	40.6
30–39	116	34.1
40–49	52	15.3
50–60	34	10.0
Marital status		
Single	178	52.4
Married	132	38.8
Widow/separate	30	8.8
Education		
Diploma	10	2.9
Bachelor’s degree or higher	330	97.1
Non-communicable diseases		
Presence	46	13.5
Absence	294	86.5
Job positions		
Public health practitioner	191	56.2
Public health technical officer	60	17.6
Registered nurse	54	15.9
Nurse practitioner	26	7.6
Dental public health officer	9	2.6
Income (Thai baht per month *)		
<15,000	37	10.9
15,000–30,000	207	60.9
>30,000	96	28.2
Work Experience (year)		
1–5	141	41.5
6–10	81	23.8
>10	118	34.7
Working hour per day		
≤8	227	66.8
>8	113	33.2

* 1 USD = 31.19 Thai baht (Accessed on 10 February 2026).

**Table 2:** Occupational hazard exposures and social support levels among healthcare personnel in SHPHs (*n* = 340).

Items	Levels: *n* (%)		
	Low	Moderate	High
Hazards exposure Overall	83 (24.4)	257 (75.6)	0 (0.0)
Physical exposure	255 (75.0)	83 (24.4)	2 (0.6)
Biological exposure	90 (26.5)	88 (25.9)	162 (47.6)
Chemical exposure	133 (39.1)	87 (25.6)	120 (35.3)
Ergonomics exposure	16 (4.7)	303 (89.1)	21 (6.2)
Psychosocial exposure	211 (62.1)	126 (37.0)	3 (0.9)
Social supports	0 (0.0)	91 (26.8)	248 (73.2)

**Table 3:** QWL levels among healthcare personnel in SHPHs by domain (*n* = 340).

Domains	QWL Levels: *n* (%)		
	Poor	Moderate	Good
Overall	11 (3.2)	155 (45.6)	174 (51.2)
Adequate and fair compensation	94 (27.6)	101 (29.7)	145 (42.6)
Safe and healthy working conditions	5 (1.5)	77 (22.6)	258 (75.9)
Opportunity to use and develop human capacities	3 (0.9)	97 (28.5)	240 (70.6)
Opportunity for continued growth and security	2 (0.6)	117 (34.4)	221 (65.0)
Social integration in the work organization	3 (0.9)	89 (26.2)	248 (72.9)
Constitutionalism in the work organization	1 (0.3)	108 (31.8)	231 (67.9)
Work and total life space	10 (2.9)	114 (33.5)	216 (63.5)
Social relevance of work life	1 (0.3)	83 (24.4)	256 (75.3)

**Table 4:** Stepwise linear regression analysis of predictors of QWL (*n* = 340).

Model		QWL					
		R^2^	B	SE	Beta	t	*p*
1	(Constant)		46.863	3.891		12.045	<0.001
	Social support	0.642	3.021	0.123	0.802	24.652	<0.001
2	(Constant)		44.931	3.878		11.586	<0.001
	Social support		2.923	0.124	0.776	23.527	<0.001
	Working hours per day	0.652	3.750	1.128	0.110	3.324	0.001
3	(Constant)		44.516	3.867		11.512	<0.001
	Social support		2.975	0.126	0.790	23.521	<0.001
	Working hours per day		3.717	1.123	0.109	3.309	0.001
	Work experience	0.655	−0.111	0.056	−0.064	−1.972	0.049

Note: Variables were selected using stepwise multiple linear regression after bivariate screening (*p* < 0.25). Only predictors retained in the final model are interpreted.

## Data Availability

The original contributions presented in this study are included in the article. Further inquiries can be directed to the corresponding authors.

## Supplementary Materials

The following supporting information can be downloaded at: https://www.mdpi.com/article/10.3390/ijerph23020272/s1, Table S1. Correlation Matrix of Study Variables Related to Quality of Working Life (QWL).

## Abbreviations

The following abbreviations are used in this manuscript:

NCDs	Non-communicable diseases
QWL	Quality of Working Life
SE	Standard Error
SHPH	Sub-district Health Promoting Hospital
