# Supplementary material for: Occupational Hazards, Social Support, and Quality of Working Life in Sub-District Health Promoting Hospitals in Southern Thailand: A Cross-Sectional Study
Source: Int J Environ Res Public Health. 2026 Feb 23;23(2):272. doi: 10.3390/ijerph23020272 (PMC12941247; doi:10.3390/ijerph23020272)
Supplement: Supplementary file 1 [file ijerph-23-00272-s001.zip › ijerph-4079011-supplementary.pdf]

**Table S1.** Correlation Matrix of Study Variables Related to Quality of Working Life (QWL).

| Variables | QWL | Gender | Age | Marital status | Education | NCDs | Job position | Income | Work experience | Working hour per day | Social support |
|---|---|---|---|---|---|---|---|---|---|---|---|
| **Gender** (Male/Female) | 0.046 | 1.000 | | | | | | | | | |
| **Age** | 0.156** | -0.032 | 1.000 | | | | | | | | |
| **Marital status** (Single, widow, separate/Married) | 0.053 | -0.055 | 0.376*** | 1.000 | | | | | | | |
| **Education** (Diploma/Bachelor degree or higher) | -0.005 | 0.092* | -0.050 | -0.003 | 1.000 | | | | | | |
| **NCDs** (Absence/ Presence) | 0.173** | -0.070 | 0.277*** | 0.123* | -0.135** | 1.000 | | | | | |
| **Job position** (Nurse, Dental public health /Public health practitioner and technical officer) | -0.165** | -0.187*** | -0.282*** | -0.057 | -0.024 | -0.058 | 1.000 | | | | |
| **Income** (<=30,000/>30,000) | 0.194*** | -0.061 | 0.790*** | 0.327*** | -0.007 | 0.229*** | -0.429*** | 1.000 | | | |
| **Work experience** | 0.106* | -0.041 | 0.956*** | 0.367*** | -0.046 | 0.274*** | -0.256*** | 0.782*** | 1.000 | | |
| **Working hour per day** (<=8/ >8) | 0.293*** | 0.010 | 0.057 | -0.041 | 0.012 | 0.104* | -0.176** | 0.029 | 0.035 | 1.000 | |
| **Social support** | 0.802*** | 0.083 | 0.253*** | 0.121* | -0.039 | 0.172** | -0.218*** | 0.276*** | 0.210*** | 0.236*** | 1.000 |
| **Hazard exposure** | -0.085 | -0.067 | -0.237*** | -0.081 | 0.011 | 0.011 | 0.025 | -0.107* | -0.210*** | 0.059 | -0.139** |

*<0.05, **<0.01 **<0.001

Note: Pearson's product–moment or point-biserial correlations were used as appropriate. The matrix was used to examine bivariate relationships and screen for potential multicollinearity prior to regression analysis.
